# Use of Social Robots in Mental Health and Well-Being Research: Systematic Review

**DOI:** 10.2196/13322

**Published:** 2019-07-24

**Authors:** Arielle AJ Scoglio, Erin D Reilly, Jay A Gorman, Charles E Drebing

**Affiliations:** 1 Social & Community Reintegration Research Program Edith Nourse Rogers Memorial Veterans Hospital Bedford, MA United States

**Keywords:** social robotics, socially assistive robots, mental health, interventions

## Abstract

**Background:**

Technology-assisted clinical interventions are increasingly common in the health care field, often with the proposed aim to improve access to and cost-effectiveness of care. Current technology platforms delivering interventions are largely mobile apps and online websites, although efforts have been made to create more personalized and embodied technology experiences. To extend and improve on these platforms, the field of robotics has been increasingly included in conversations of how to deliver technology-assisted, interactive, and responsive mental health and psychological well-being interventions. Socially assistive robots (SARs) are robotic technology platforms with audio, visual, and movement capabilities that are being developed to interact with individuals socially while also assisting them with management of their physical and psychological well-being. However, little is known about the empirical evidence or utility of using SARs in mental health interventions.

**Objective:**

The review synthesizes and describes the nascent empirical literature of SARs in mental health research and identifies strengths, weaknesses, and opportunities for improvement in future research and practice.

**Methods:**

Searches in Medline, PsycINFO, PsycARTICLES, PubMed, and IEEE Xplore yielded 12 studies included in the final review after applying inclusion and exclusion criteria. Abstract and full-text reviews were conducted by two authors independently.

**Results:**

This systematic review of the literature found 5 distinct SARs used in research to investigate the potential for this technology to address mental health and psychological well-being outcomes. Research on mental health applications of SARs focuses largely on elderly dementia patients and relies on usability pilot data with methodological limitations.

**Conclusions:**

The current SARs research in mental health use is limited in generalizability, scope, and measurement of psychological outcomes. Opportunities for expansion of research in this area include diversifying populations studied, SARs used, clinical applications, measures used, and settings for those applications.

## Introduction

### Overview

There is a well-documented gap between individuals in need of support for mental health concerns and those who receive care [1-4]. To address the treatment disparity between individuals in need of psychiatric care and those who receive it, the field of mental health has expanded from offering exclusively in-office clinical care, including interventions such as psychotherapy, care management, and medication, to offering treatment in a wide array of settings and with varied interface platforms. The use of technology in providing at-home care options have identified both potential [5] and challenges [6].

One study found that technology-assisted support and treatments may be appealing to potential patients and health centers because telehealth delivery methods can improve access and are often cost effective [7]. There are multiple reasons why individuals may not be able to access needed care, including lack of available therapists [8], lack of transportation [9], stigma around engaging in mental health care [10], and financial barriers [11]. Mobile intervention options can also be used to extend in-person clinical treatments such as cognitive-behavioral therapy for insomnia or posttraumatic stress disorder treatments to the home [12,13]. Similarly, online interventions have the potential to reach patients who might otherwise not have access to mental health or other clinical care, as 82% of adults in the United States have access to either the internet at home or wireless mobile devices [14]. At-home technology platforms have attempted to meet the needs of such persons, to either replace or supplement in-person treatments [5,15].

Following initial program development, developers often purport that technology-supported behavioral interventions can be more easily implemented than in-person options, without the limitations of travel, local resources, training new practitioners to implement a treatment model, or monitoring treatment fidelity [16]. However, creating and implementing such systems can be difficult. For instance, real-world use of some mHealth tools by clinics and consumers remains low [17] even after the tools have been assessed for usability issues. At the same time, research has shown that mHealth tools may help clients self-manage their own treatment and goals across multiple diagnoses related to chronic physical and mental health challenges using apps, clinical portals, and texting interfaces [18,19]. Some research suggests that the utilization gap between developed technologies and their intended consumers may be related to engagement [20].

Although initial research appears promising, mHealth technology has documented limitations regarding treatment engagement [21,22]. Current mHealth technologies often rely on intervention strategies with minimal personalization and interactions, including mobile phone apps and one-way texting, which lack key factors of mental health interventions: real-time interactive engagement, simple user experiences, transdiagnostic capabilities within one platform, and personalized feedback [23]. Only recently has there been a push to develop and assess how socially interactive technologies, such as computer-animated virtual therapists, can be leveraged for mHealth interventions to support accountability, provide continuously tailored feedback, and form a social relationship to successfully impact client wellness [24]. Consequently, there is both a need for and room to improve the mHealth platforms used in mental health interventions to create a more client-centered and engaging experience.

### Social Robots and Well-Being

To address engagement and motivational difficulties with mobile mental health interventions, researchers have begun to explore the possibility of using animated characters and social robots as personalized social companions to deliver or supplement behavioral interventions [25,26]. Socially assistive robots (SARs) are robotic technology platforms with audio, visual, and movement capabilities. Their purpose is to create friendly and effective interaction with a human user with the additional aim of giving assistance to the user and achieving measurable progress in quality of life, often related to motivation, rehabilitation, or learning [27]. SARs are embodied, taking up physical space in the world and not merely existing on a screen, and can use audio and/or closed captioning to converse socially with humans, depending on their design [28]. It is important to note that SARs are both platforms for interventions and also interventions in and of themselves; they can learn and engage socially with individuals while also presenting interventions to users similar to mobile apps (eg, skills training, health tracking). They can engage users across multiple sensory options, most often including sound, sight, and touch, which can create multiple modalities for the delivery of content or interactions, depending on user preferences or personal physical abilities [29]. Given their multiple abilities, SARs may potentially integrate traditional app- and telehealth-related supports with an interactive social companion, providing a more engaging and responsive platform for users.

Although research with SARs is still in its early stages, preliminary research has reported positive participant responses to SARs assisting in physical health interventions related to increasing exercise with the elderly [30], improved cardiac rehabilitation through self-reported usefulness of SARs to assist in completion of rehabilitation tasks [31], and improved medication management through medication reminders by an SAR [32]. These specific robots were created to serve as embedded reinforcers of tasks, health behaviors, and prosocial interactions and are used across a wide range of conditions. One study found that SARs may assist with weight management, motivation, and self-monitoring strategies, with engagement sustained beyond what has been found with the same treatment delivered passively online [26].

There is limited evidence that SARs can assist with mental health and well-being interventions in pediatric populations by providing comfort or coaching [33,34]. Additionally, a 2013 meta-analysis focusing on the psychological outcomes of robot-enhanced therapies suggests that social robots could be used as a complementary tool in therapy for specific populations, particularly with children [35]. The function of social robots in adult populations is different and should be studied separately; children and adolescents often respond differently to robots, and the focus should be on developmental and skill-based learning (eg, to support children’s play [36] or assist adolescents with autism [37]). However, these platforms and their socially responsive capabilities can be modified to assist adults with their mental and behavioral health goals, given the dual nature of social robots to create personalized, affective relationships with users and assist in setting, tracking, and supporting users in meeting specific goals [38]. Unfortunately, little is known about the nature of social robots and their potential use in assisting in the psychological well-being of adult populations.

Social robots have been used in research with children, usually involving children with developmental disabilities [39]. However, the function of social robots is different in pediatric versus adult populations. Research with children focuses on robots as models of appropriate behavior or physical helpers with manual tasks [40]. There is more variability in the functionality of social robots in research with adults, thus we restricted our search to adult populations.

There is room for expansion in the use of SARs in mental health research with adults and how the research into robots and mental health has developed in recent years given the fast-paced nature of robotics. Reviewing the existing empirical literature on the use of social robots for mental health interventions is essential to determine the current state of the research and make suggestions regarding future areas for investigation. This systematic review of the literature attempts to synthesize studies using social robots to affect mental health and psychological well-being outcomes, identify the current strengths and weaknesses in the research, and suggest opportunities for growth and exploration.

## Methods

This study is a systematic synthesis of the literature from the past 10 years that examines the use of social robots in mental health and psychological well-being. The fields of robotics and artificial intelligence are rapidly changing and this review is meant to reflect the current research in this area. We sought to answer the question: How have social robots been used to enhance mental health services for adults? A search was conducted on June 20, 2018, in the databases Medline, PsycINFO, PsycARTICLES, PubMed, and IEEE Xplore. We used several search term combinations to search titles, abstracts, keywords, and text in articles: social robot* + mental health, social robot* + counseling, social robot* + therapy, social robot* + psychotherapy, socially assistive robot* + mental health, socially assistive robot* + counseling, socially assistive robot* + therapy, socially assistive robot* + psychotherapy. Search terms were developed in consultation with coauthors and a research librarian. In our search, we excluded studies published in languages other than English, studies published prior to 2008, and studies that focused on pediatric populations. This search yielded 48 articles in total for abstract review after removing duplicates (n=9). Exact search strings for each database are included in Multimedia Appendix 1.

**Figure 1:**
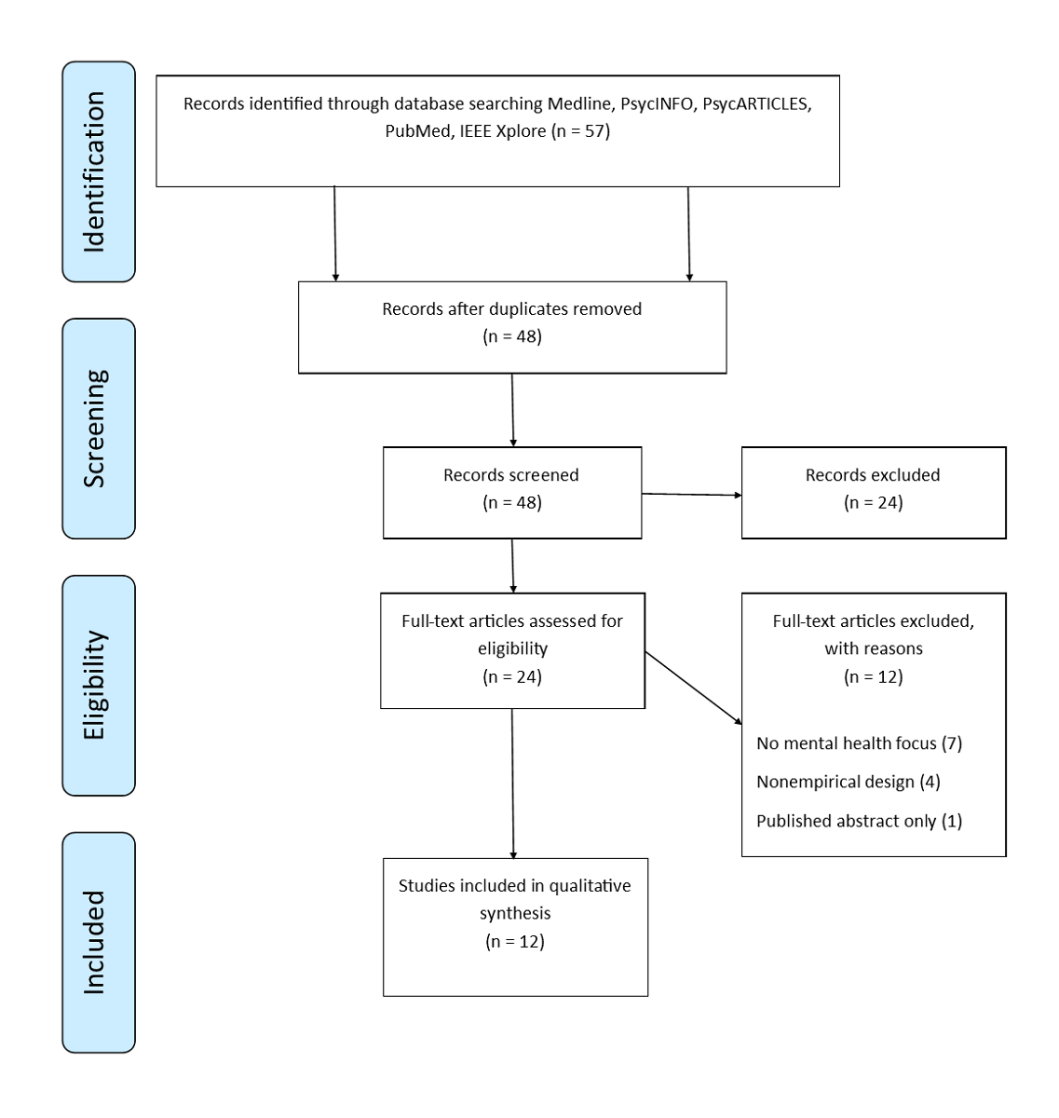
Preferred Reporting of Systematic Reviews Search and Review diagram.

The authors used Covidence.org to organize the review and conduct blinded abstract and full-text reviews. This review is not formally registered. Two authors independently reviewed all abstracts and met to come to consensus on the inclusion or exclusion of articles in conflict. The inclusion criteria were that the article was an empirical study involving data collected on the direct interactions between a human participant (aged 18 years or older) and a social robot (an embodied robotic platform meant to form an assistive or affective connection with users) and that the authors explicitly stated their study focused on a mental health treatment population (eg, psychiatric patients) or used a mental health–focused intervention (eg, motivational interviewing). Included studies had to report on one or more mental health or psychological well-being outcomes with data collected to measure the robot’s possible relationship with the mood, psychological welfare, or comfort of users. Although our search process did not include the term psychological well-being, it became clear in our review process that articles that explicitly aimed to examine mental health actually measured aspects of well-being rather than specific mental health constructs. Thus, we expanded our inclusion criteria to include measurement of a psychological well-being outcome. Twenty-four articles were excluded in the abstract review process, detailed in Figure 1. There was a discrepancy on rating abstracts with 2 articles that was resolved through consensus agreement, indicating good interrater reliability. This left 24 articles for full-text review. The same two authors independently reviewed all 24 full-text articles and met to resolve any conflicts by consensus agreement. Twelve articles were excluded in the full-text review phase because they did not use an empirical design (eg, it was a theoretical or commentary paper) or the focus of the study was not on mental health or well-being (eg, mental health was not a primary outcome). Twelve studies remained for inclusion in our final review.

Data were extracted from the 12 articles included in the final review. Specifically, detailed information about the sample size and characteristics of the population, study design, mental health or well-being outcome and measurement, robot used, intervention implemented, study findings, and possible biases were all recorded. Details about definitions of mental health or well-being outcome and purpose of the intervention were also identified. Studies were assessed for methodological quality, but quality assessments were not used to exclude any studies. Instead, quality assessments served to identify consistent weaknesses across studies. Contact with individual study authors was ultimately not deemed necessary to extract needed information from the included studies.

## Results

### Summary

The 12 studies included in our review used 5 different social robots (Paro, NAO, CRECA, Betty, and Haptic Creature) constituting 3 major areas of robotic applications for mental health developed from our review: comfort/companionship, stress reduction, and motivation. The studies were published between 2010-2018 in a variety of peer-reviewed journals; 4 studies were published conference papers. Sample sizes for the studies ranged from 2 to 248; 7 of the 12 studies were conducted in elderly populations in nursing home settings, 2 were conducted with college students, 2 with hospital staff, and 1 with women aged 19 to 45 years recruited from the community. A minority of studies (n=3) focused on participants who were aged 45 years or less. Three studies were not conducted with a clinical mental health sample but from participants recruited from the community or from local colleges. These studies were included in the review because they measured mental health or well-being outcomes such as self-reported anxiety or stress reduction. Table 1 describes the studies, samples, interventions, and main findings with respect to the effects of social robots on psychological outcomes collected in our review.

**Table 1:** Main evidence from systematic review.

First author, year	Study design	Sample size and characteristics	Robot	Mental health or well-being outcome	Intervention	Main findings
Bemelmans, 2015 [41]	Pre-post (single session)	71 nursing home residents with dementia (age range not reported)	Paro	IPPA^a^ score and mood via COOP/WONCA^b^ chart recorded by care provider	Quasi-experimental time series study: 15-minute interaction with Paro while experiencing unrest or negative mood	Significant positive effect on mood and IPPA score
Galvão Gomes da Silva, 2018 [42]	Pre-post (single session)	20 psychology students (aged 18 to 62 years, majority under 25 years)	NAO	24 open-ended self-reported items in questionnaire assessing motivation for exercise (author created)	Two lab sessions of motivational interviewing for exercise with NAO (1-week interval between sessions)	Positive appraisals of robot as nonjudgmental Increased “change talk” in participants Increased motivation to exercise
Kurashige, 2017 [43]	Pre-post (single session)	12 male students aged 21 to 23 years (mean age not reported)	CRECA^c^	Author-created self-report items (15) on conversational flow, perceived trust/reliability in CRECA, and stress reduction	Motivational interviewing session with nodding or not nodding CRECA around stress management	Positive appraisal of dialogue with nodding CRECA Self-reported reduction in anxiety
Lane, 2016 [44]	Pre-post	106 VA^d^ community living center elderly patients (aged 58 to 97 years, mean age 80 years)	Paro	Care staff observed behaviors and mood on researcher-created tracking sheet across 3 time periods (baseline, Paro treatment, posttreatment)	Veteran was actively presented with or observed to be actively using Paro for a minimum of 5 minutes	Observed that Paro reduced negative behavior and mood states Observed that Paro induced increases in indicators of positive mood states
Loi, 2017 [45]	Pre-post	45-bed unit for younger adults with neuropsychiatric conditions, (residents < 65 years, mean age not reported)	Betty	Staff completed a pre- and post-SARs^e^ questionnaire regarding patient well-being, enjoyment, and quality of life (items based on technology acceptance model)	Betty was present at the facility for 12 weeks; engaged with residents via conversations, music, relaxation exercises, and games	Staff reported that Betty was helpful to patients by being comforting, relaxing, and improving the well-being of residents
Moyle, 2018 [46]	Cluster randomized RCT^f^	Residents with dementia in a long-term care facility (mean age not reported)	Paro	Staff reported patient comfort and well-being (qualitative interview)	Three 15 minute interactions were observed between Paro and elderly residents within 3 treatment groups: Paro, plush toy, or usual care	Staff indicated there were benefits to using Paro as a companion to elderly patients, although Paro did not comfort all residents
Šabanović, 2013 [47]	Pre-post	10 nursing home residents with dementia (ages not reported)	Paro	Researcher videotaped and coded interactions based on positive engagement with others	Residents interacted with Paro over 7 weekly sessions	Observed an increase in prosocial interaction between residents
Sefidgar, 2016 [48]	Pre-post (single session, within-subject design)	38 women aged 19 to 45 years, mean age 23.8 years	Haptic Creature	Self-reports on the SAM^g^; STAI-6^h^	Interaction with Haptic Creature on lap, compared with nonmoving stuffed animal replica	Biometric indicators of relaxation related to heart and respiration rates significantly decreased relative to stroking a nonbreathing replica Participants reported feeling calmer and happier
Valentí Soler, 2015 [49]	Pre-post	211 nursing home patients with dementia, 37 at day care facility (total n=248; age range 58 to 100 years, mean age 84.7 years	Paro, NAO	Staff reported on the Apathy Inventory and QUALID^i^ scale	Comparing interactions with Paro, NAO, and live dog over 3 months	Apathy and irritability improved for NAO and Paro groups Quality of life improved for Paro group
Wada, 2010 [50]	Pre-post	2 elderly individuals and 1 caregiver, age not reported	Paro	Researcher observed emotional responses and behaviors (ie, smiling)	Caregivers engaged in a manual-assisted 30-minute interaction between residents and Paro (4 sessions)	Observed-recorded increase in positive behaviors in 1 participant (smiling, laughing), no significant change in other participant
Wada, 2012 [51]	Pre-post	12 elderly participants (mean age 86.8 years) and 9 caregivers (mean age 28.1 years)	Paro	Observation sheet recording participant behaviors and emotional reactions (researcher-recorded)	Manual-assisted interaction with Paro; observed before caregiver used manual and after caregiver used manual	The manualized Paro interaction increased contentment and positive social interactions
Wada, 2014 [52]	Pre-post	64 elderly individuals in 7 elder-care facilities (mean age 86.5 years)	Paro	Observation sheet recording perceived participant behaviors and mood (anxiety, depression, aggression)	Manual-assisted interaction with Paro over 5 months	Following Paro interactions, caregivers observed decreases in perceived anxiety, depression, or aggression in 25 residents (39%)

^a^IPPA: Individually Prioritized Problems Assessment.

^b^COOP/WONCA: Primary Care Cooperative Information Project/World Organization of Colleges, Academies, and Academic Associations of General Practitioners/Family Physicians.

^c^CRECA: Contextual Respectful Counseling Agent.

^d^VA: US Department of Veterans Affairs.

^e^SAR: socially assistive robot.

^f^RCT: randomized controlled trial.

^g^SAM: Self-Assessment Manikin.

^h^STAI-6: State-Trait Anxiety Inventory.

^i^QUALID: Quality of Life in Late Stage Dementia.

### Robotic Devices

**Figure 2:**
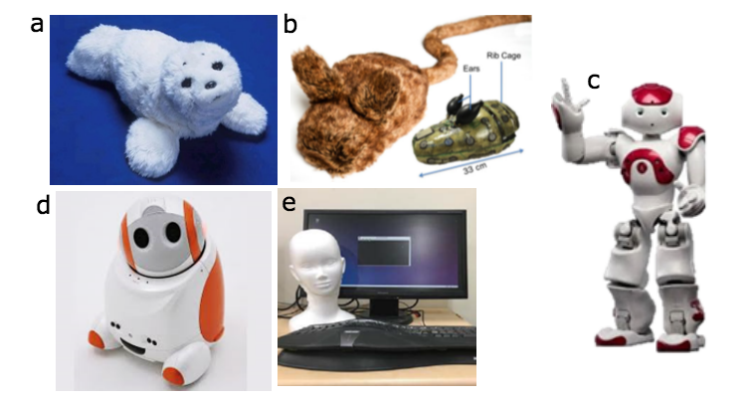
Social robots used in reviewed articles: (a) Paro, AIST [41]; (b) Haptic Creature [48], photo by Martin Dee; (c) NAO, Aldebaran Robotics; (d) Betty [45]; and (e) CRECA [43].

A majority of studies (n=8) used Paro, a robot that resembles a baby harp seal, with 2 studies using NAO, and 1 study using CRECA (Contextual Respectful Counseling Agent), Haptic Creature, and Betty. In Figure 2 and Table 2, pictorial and text descriptions of the 5 social robots are provided. Overall, the appearance of the robots used matched their purpose, with Paro and the Haptic Creature resembling animals, as the researchers aimed to use audio, visual, and tactile sensors to mimic animal-assisted therapy. The humanoid SARs (CRECA, Betty, and NAO) had audio, visual, and tactile sensors as well but also used additional sensors to communicate verbally with users to provide interactions related to relaxation or mental health treatment (ie, counseling, motivational interviewing). Although all the robots were capable of limited movement, only NAO was able to walk and assume a standing position if needed. The weight of the robots varied greatly, with the heaviest weighing 14 lbs (Betty) and the lightest weighing 6 lbs (Paro; information on height/weight of CRECA not available).

**Table 2:** Description of social robots used in reviewed articles.

Robot	Physical appearance and specs	Sensors	User interactivity	Purpose
Paro	Paro is a robotic harp seal, weighing 6 lbs and 22.4 inches long. Paro can be recharged through its “pacifier” battery charger. Its fur is removable, washable, fluffy, and antibacterial. The US Food and Drug Administration has classified Paro as a “biofeedback medical device,” and the platform is not programmable by external users.	Has five kinds of sensors: tactile, light, audition, temperature, and posture sensors, with which it can perceive people and its environment.	He can sense when being touched by its tactile sensor, or when being held by a posture sensor. Can also recognize the direction of voice and words such as its name, greetings, and praise with its audio sensor. His voice imitates a harp seal.	Paro is meant to provide similar comfort as animal therapy for patients in facilities where live animals may present treatment or logistical difficulties. Paro may be used for comfort, companionship, or stress reduction.
Betty	Betty is an individualized, socially-assistive robot, with the technological name Partner Personal Robot PaPeRo. Betty is 15.35 inches tall and weighs about 14 lbs. Betty is programmable by users external to the company.	Betty has audio, touch, movement, and visual sensors; specifically, it is equipped with a camera, microphone, a touch-pad, and speakers.	Voice recognition is the primary modality for interacting with Betty. The robot can also make human-like gestures, has voice recognition capabilities, is mobile, and can be programmed with a person’s preferences (eg, books, games, or music).	Betty may be used for motivation, entertainment, or companionship. The robot is meant to provide human-like interactions and reciprocal engagement, while also providing a calming effect for users.
NAO	NAO is brightly colored with large eyes and humanoid appearance. NAO stands at 22.8 inches tall and weighs 12.1 lbs. Its default walking speed is 0.2 mph. The robot is fully programmable.	It has multiple sensors for touch, sound, speech, and visual recognition. NAO is also capable of movement, with both fall and fall recovery capabilities.	NAO interacts with users via an audio system, often with accompanying movements and lights. It has speech recognition and dialogue with NAO is available in 20 languages.	It has been used in research with children who have developmental disorders or disabilities. NAO is also used for motivation or companionship
Haptic Creature	The Haptic Creature is a comfort robot and was designed based on human-animal interaction models. It is characterized as an expressive animatronic lap-pet (size of a large cat). It is 12.9 inches long and weighs 5.5 lbs. The platform utilizes custom programming that may be available to external programmers upon request.	Includes a 30-item touch dictionary developed from social psychology and human-animal interaction literature. It perceives movement and touch, responding with ear stiffness, modulated breathing, and vibrotactile purring.	Users interact with the Haptic Creature solely through touch, with the robot responding with movement and visual cues to mimic relaxed breathing.	Through touch, it promotes emotional interaction with the user with the aims to reduce anxiety similar to animal assisted therapy. It can also be used for comfort or stress reduction.
CRECA	CRECA stands for “Context Respectful Counseling Agent” and works in conjunction with an on-screen counseling agent avatar. The platform utilizes custom programming that may be available to external programmers upon request	This robot is connected to a computer and microphone to perform speech functions using natural language processing. It can also perform nodding movements.	It can converse with the users, respond to client verbalizations with prompts for continued discussion, and nod its head to validate the user’s responses.	CRECA primarily serves as an educational or motivational robot that can mimic the verbal and non-verbal interactions between counselors and clients

### Study Interventions

The reviewed studies had 2 types of interventions and varied in robotic-interaction frequency. Four studies conducted a single-time lab research session to assess their SARs [41-43,48], while the majority (n=8) embedded their SARS within a facility over a specified period of time to evaluate the impact of their SARs on residents. None of the studies employed a robot within the personal home of a participant. Although inclusion criteria were meant to capture SAR research with a mental health focus and on persons with identified mental health issues, no studies were conducted specifically with persons reporting psychological diagnoses.

In the reviewed studies, the SAR was used as the intervention, and participants were assessed to see if interacting with the robot resulted in changes in well-being or mental health. In addition, although all of the studies used at least one social robot as the main platform for a well-being or mental health intervention, one [49] used two robots in order to compare either a live animal, Paro, or NAO as the mode of delivering comfort to nursing home residents with dementia. In addition, 3 studies [43,46,48] used less responsive comparisons as controls for their SARS, including a plush animal comparison (n=2) or a nonnodding CRECA (n=1). A minority of studies (n=2) used an SAR as a platform to conduct motivational interviewing sessions with social robots, one focused on motivation to exercise using NAO and the other focused on stress reduction using CRECA [43,48]. The remaining 10 research studies focused on the social robot as a means of comforting, increasing positive emotions, or providing companionship to participants, with 9 of these using the robots within the context of elderly or long-term care settings.

### Study Design and Measurement

Our review revealed that 6 of the studies used clinical care staff to observe and measure outcomes related to mood and mood-related behavioral changes, 3 used self-report data [42,43,48], and 3 had researchers use tracking sheets [44,46,50] to report on mood and mental health outcomes. These methods may be an artifact of the populations studied, due to the majority of studies involving persons with cognitive issues who would make self-report challenging. In addition, only 4 studies [41,46,48,49] used nonresearch-created questionnaires, using previously validated measurements such as the Apathy Inventory and Quality of Life in Late Stage Dementia scale. Although one study [46] used an RCT (randomized controlled trial) study design, most of the studies (n=11) used a pre-post design, measuring changes in psychological well-being or intervention impact before and after interacting with a SAR.

### Mental Health Outcomes

Overall, results regarding the impact of social robot–delivered mental health interventions and interactions ranged from generally positive to mixed. The majority of studies focused on symptom reduction related to mood and positive quality of life changes after robot interactions. The majority (n=11) reported positive increases in mood, comfort, or stress reduction following the social robot interventions, although 2 [46,50] showed mixed results on whether Paro comforted elder-care residents. For the 7 studies that focused on elderly and dementia populations, outcomes included observed aggression, contentment, anxiety, and depression [41,44,45,49-52]. However, nearly all studies had a main goal of assessing feasibility or usability of the social robot in a given population. Since mental health outcomes were secondary and these studies were pilot studies, psychometrically validated measures were seldom used and instead measures were frequently created for the particular study.

Two studies reported on functional outcomes related to quality of life. These studies focused specifically on physical health and well-being, with one reporting a generally positive impact on exercise [42] and one reporting a reduction in physical indicators of stress and an increase in self-reported mood after interaction with a social robot [41]. Finally, 2 studies assessed the ways in which social robots facilitate positive social interactions, with one reporting increased social interaction among participants in a nursing home and their caregivers following an interaction with Paro [51] and the other noting that social interactions among residents in a nursing home increased after Paro was integrated into the facility [47].

## Discussion

### Principal Findings

Overall, our review revealed the nascent nature of mental health research with social robots. Although there is a rising interest in using social robots in psychological interventions, there is still a very modest research base examining this application. Our 12 reviewed studies included the use of 5 distinct social robots to influence various mental health or well-being outcomes. The majority focused on providing comfort and companionship to study participants (Paro, Haptic Creature). A minority of studies used SARs to implement a specific intervention (eg, motivational interviewing with NAO and nodding CRECA). The impact of social robot–delivered mental health interventions and interactions ranged from generally positive to mixed, with some studies finding positive changes in mood and quality of life after robot interactions.

This review suggests that existing studies of the potential impact of SARs on psychological well-being are limited in generalizability, scope, and measurement. Specifically, nearly all of the studies conducted in this area have occurred in elderly care facilities or laboratory settings, with a bimodal distribution of participants ranging from quite young (under 24) to elderly populations within nursing home facilities (often over 65 years). Our findings are consistent with a previous review of SAR use in care of the elderly [35]), which found studies in this area to be methodologically limited, so much so that even optimistic findings required additional replication prior to making clear conclusions about SAR effectiveness. This also relates to our finding that the majority of reviewed studies used clinical care or researcher-reported outcomes. Specifically, since multiple studies were conducted with elderly samples suffering from dementia or neuropsychiatric conditions, observational data may have been considered most feasible and more valid than self-report data. Similarly, surveying caretakers of impaired elderly persons may have been more feasible than surveying the patients themselves. The lack of validated scales used in the studies underscores the nascent state of this field and the need for further and more structured research.

Social robot–delivered interventions may constitute a promising treatment for chronic conditions and health management needs in elderly populations based on the findings of studies included in this review. In particular, the available data indicate that Paro may be a useful tool for increasing socialization and decreasing aggression in dementia populations. However, there is insufficient evidence that this finding can be generalized to other populations or even to nursing home residents without dementia. In addition, some studies identified were conference paper proceedings, which highlights a limitation of the research. Conference papers are not necessarily peer reviewed and may indicate a higher risk of bias.

### Limitations

Generalizability of these findings is also limited by the study characteristics. Many of the studies had very small sample sizes, which also limits the generalizability of findings. Moreover, included studies frequently had very brief interventions with simple pre-post study designs, which might make it difficult to assess differences in pre and post data and preclude conclusions about the efficacy of the interventions. We did not include specific mental health diagnoses in our searches, and therefore some studies using SARs to target very specific diagnoses, rather than mental health more generally, may have been excluded. In addition, we did not include papers that focused only on the development of a particular SAR because we only included studies that had participants. There is also a lack of definitional clarity in the concept of social robots in general, and this may lead to different conceptualizations of SARs in the literature.

There is a clear need for greater testing and programming of robots to assist with patient care within the home. Although many of the research studies reported a future aim of using social robots within the home for mental health support and interventions, no study had currently begun this level of at-home technology testing. Without such testing, it is unclear whether an at-home mental health companion, coach, or motivator would have a strong positive effect on the quality of life of patients managing their depression, anxiety, or other psychiatric conditions where health happens most—in the home. Choosing the appropriate SAR to use for future research should entail a review of not only this research but also the robot characteristics necessary for the user. For instance, many of the robots reviewed weighed more than 10 lbs, which can be contraindicated with certain populations should they need to lift the SAR. Such considerations are imperative in order to align an intervention’s purpose, user, and chosen robotic platform.

Methodologically, there is room for improving and extending the current research base of social robots in mental health interventions. The results of this review show that nearly all studies in this area are preliminary or pilot studies, and few include validated measures of mental health outcomes. Often, the primary aim of such research is usability, feasibility, and acceptability of the social robots with mental health measures secondary to this main goal. Mental health outcomes were often vaguely defined as the observed reduction of anxiety or negative mood symptoms. Expanded use of self-report questionnaires or clinician-administered measures with psychometric validation is indicated. In addition, outcomes should be aligned with what patients might care about in treatment outcomes, which may include an increase in functional abilities, social interactions, or self-reported quality of life. Nearly all of the studies reviewed used single-session robot interactions that may make tracking changes in mental health difficult. As previously stated, many studies included participants with cognitive difficulties, which might make self-report assessments difficult or impossible, hence their use of observed mental health outcomes. However, if the purpose of social robot–delivered treatments is to capitalize on the functionality of such robots—neutral, asynchronous, always available, and capable of personalized tracking and feedback—such testing and adequate measurement is essential to the creation of patient-centered social robots. Because of the heterogeneity in how outcomes were reported, we could not perform a meta-analysis or draw conclusions about possible biases at work across studies.

### Practical Implications and Future Research

After reviewing the existing research on social robots and mental health, it is clear that there are ample opportunities to test and measure the ways social robots could be useful adjuncts to mental health treatments for various adult populations. Current research has used mental health outcomes as a secondary focus, and future research should explore the potential benefits of SARs in specific clinical populations with difficulty accessing care. Examples of such populations might include veterans living with chronic pain in rural areas, individuals with mental health needs who cannot make appointments during regular business hours, individuals with transportation issues, or individuals who feel stigmatized in traditional mental health care settings. Exploration of motivational, companionship, and social facilitation functions of SARs were assessed in a minority of studies. In Table 3, we highlight further recommendations and areas of consideration for future research into the mental health and clinical applications of social robots.

**Table 3:** Considerations and recommendations for future research.

Research considerations	Recommendations
Internal validity	Improve upon and expand methods beyond pilot studies Use validated mental health outcome measures when advancing beyond pilot feasibility studies Account for potential mediators between socially assistive robot interactions and mental health outcomes, such as usability or technology issues
External validity/generalizability	Expand beyond dementia and developmentally disordered populations to include a range of ages and diagnoses (with special attention to those who may not currently have access to needed care) Explore use of socially assistive robots across different settings, from medical facilities to at-home robots
Inclusion of theory	Use existing literature on human-robot interactions to account for study aims and interventions design decisions Embed psychological theory into future research—such as object relations—to explore individual mental health outcomes and reactions and perceived efficacy of socially assistive robots
Dissemination and translation	Expand future research to robots that can engage in more human-like social interaction Consider close, multidisciplinary collaborations (eg, between clinicians, researchers, and robotics programmers) to allow for iterative and responsive intervention development
Cultural concerns	Investigate the impact of sociocultural beliefs and differences related to technology comfort, linguistic challenges, and interest in socially assistive robots for mental health Focus on specific mental health populations that might be uniquely suited to benefit from socially assistive robots

### Conclusions

Our review sought to examine how social robots have been used to influence mental health in general, and a possible limitation of our review is that we chose not to include specific mental health terms (eg, depression) in our search process. In addition, since our search, which focused on mental health, yielded studies that mostly assessed aspects of well-being, it is possible that including multiple more specific mental health terms might have yielded different results.

Overall, better integrating and expanding on the mental health implications of social robots will clearly complement the ongoing drive in the field of psychology to better assist clients at home with supportive exercises, goal tracking, and an asynchronous care option. Although our review revealed that the use of SARs in mental health research is not yet widespread, new robots and programming are constantly changing, adapting, and expanding. The use of SARs in mental health research and mental health interventions is nascent and has thus far been restricted to specific populations with limited measurement and scope. There is an abundance of opportunity in this area for growth, expansion, and exploration to triangulate SARs usability and efficacy data as the next step in advancing this field.

## Appendix

Multimedia Appendix 1 Search strings.

## Abbreviations

COOP/WONCA: Primary Care Cooperative Information Project/World Organization of Colleges, Academies, and Academic Associations of General Practitioners/Family Physicians
CRECA: Contextual Respectful Counseling Agent
IPPA: Individually Prioritized Problems Assessment
QUALID: Quality of Life in Late Stage Dementia
RCT: randomized controlled trial
SAM: Self-Assessment Manikin
SAR: socially assistive robot
STAI-6: State-Trait Anxiety Inventory
VA: US Department of Veterans Affairs
