# Supplementary material for: Use of Social Robots in Mental Health and Well-Being Research: Systematic Review
Source: J Med Internet Res. 2019 Jul 24;21(7):e13322. doi: 10.2196/13322 (PMC6685125; doi:10.2196/13322)
Supplement: Multimedia Appendix 1 [file jmir_v21i7e13322_app1.docx]

**PsycArticles & PsycINFO:**

| Search ID# | **Search Terms** | **Search Options** |
| --- | --- | --- |
| S5 | SU ( social robot OR social robots OR socially assistive robot OR socially assistive robots ) AND SU ( mental health OR counseling OR therapy OR psychotherapy ) | **Limiters - Published Date: 20080101-20180620; Age Groups: Adulthood (18 yrs & older), Young Adulthood (18-29 yrs), Thirties (30-39 yrs), Middle Age (40-64 yrs), Aged (65 yrs & older), Very Old (85 yrs & older)**  **Narrow by Language: - english**  **Search modes** - Boolean/Phrase |
| S4 | TX ( social robot OR social robots OR socially assistive robot OR socially assistive robots ) AND TX ( mental health OR counseling OR therapy OR psychotherapy ) | **Limiters - Published Date: 20080101-20180620; Age Groups: Adulthood (18 yrs & older), Young Adulthood (18-29 yrs), Thirties (30-39 yrs), Middle Age (40-64 yrs), Aged (65 yrs & older), Very Old (85 yrs & older)**  **Narrow by Language: - english**  **Search modes** - Boolean/Phrase |
| S3 | AB ( social robot OR social robots OR socially assistive robot OR socially assistive robots ) AND AB ( mental health OR counseling OR therapy OR psychotherapy ) | **Limiters - Published Date: 20080101-20180620; Age Groups: Adulthood (18 yrs & older), Young Adulthood (18-29 yrs), Thirties (30-39 yrs), Middle Age (40-64 yrs), Aged (65 yrs & older), Very Old (85 yrs & older)**  **Narrow by Language: - english**  **Search modes** - Boolean/Phrase |
| S2 | TI ( social robot OR social robots OR socially assistive robot OR socially assistive robots ) AND TI ( mental health OR counseling OR therapy OR psychotherapy ) | **Limiters - Published Date: 20080101-20180620; Age Groups: Adulthood (18 yrs & older), Young Adulthood (18-29 yrs), Thirties (30-39 yrs), Middle Age (40-64 yrs), Aged (65 yrs & older), Very Old (85 yrs & older)**  **Narrow by Language: - english**  **Search modes** - Boolean/Phrase |
| S1 | ( social robot OR social robots OR socially assistive robot OR socially assistive robots ) AND ( mental health OR counseling OR therapy OR psychotherapy ) | **Limiters - Published Date: 20080101-20180620; Age Groups: Adulthood (18 yrs & older), Young Adulthood (18-29 yrs), Thirties (30-39 yrs), Middle Age (40-64 yrs), Aged (65 yrs & older), Very Old (85 yrs & older)**  **Narrow by Language: - english**  **Search modes** - Boolean/Phrase |

**PubMed:**

| Search **((((social robot[Text Word] OR social robots[Text Word] OR socially assistive robot[Text Word] OR socially assistive robots[Text Word])) AND (mental health[Text Word] OR counseling[Text Word] OR therapy[Text Word] OR psychotherapy[Text Word]))) NOT ((infant[mh] OR child[mh] OR adolescent[mh]) NOT adult[mh])** Sort by: **Best Match**Filters: **Publication date from 2009/01/01 to 2018/06/20; English** |  |
| --- | --- |
| Search **((((social robot[Title/Abstract] OR social robots[Title/Abstract] OR socially assistive robot[Title/Abstract] OR socially assistive robots[Title/Abstract])) AND (mental health[Title/Abstract] OR counseling[Title/Abstract] OR therapy[Title/Abstract] OR psychotherapy[Title/Abstract]))) NOT ((infant[mh] OR child[mh] OR adolescent[mh]) NOT adult[mh])** Sort by: **Best Match** Filters: **Publication date from 2009/01/01 to 2018/06/20; English** | |
| Search **(((((social robot OR social robots OR socially assistive robot OR socially assistive robots)) AND ( "2009/01/01"[PDat] : "2018/06/20"[PDat] ) AND English[lang])) AND (((mental health OR counseling OR therapy OR psychotherapy)) AND ( "2009/01/01"[PDat] : "2018/06/20"[PDat] ) AND English[lang])) NOT ((infant[mh] OR child[mh] OR adolescent[mh]) NOT adult[mh])** Sort by: **Best Match** Filters: **Publication date from 2009/01/01 to 2018/06/20; English** | |
|  |  |

**MEDLINE (in Ovid):**

|  | (((social robot or social robots or socially assistive robot or socially assistive robots) and (mental health or counseling or therapy or psychotherapy))  not ((infant or child or adolescent) not adult)).af. | | 27 |
| --- | --- | --- | --- |
| limit 1 to (english language and yr="2009 - 2018") | | 25 |  |
| ((social robot or social robots or socially assistive robot or socially assistive robots) and (mental health or counseling or therapy or psychotherapy)).ti. not ((infant or child or adolescent) not adult).af. | | 1 |  |
| limit 3 to (english language and yr="2009 - 2018") | | 1 |  |
| ((social robot or social robots or socially assistive robot or socially assistive robots) and (mental health or counseling or therapy or psychotherapy)).kf. not ((infant or child or adolescent) not adult).af. | | 2 |  |
| limit 5 to (english language and yr="2009 - 2018") | | 2 |  |
| ((social robot or social robots or socially assistive robot or socially assistive robots) and (mental health or counseling or therapy or psychotherapy)).ab. not ((infant or child or adolescent) not adult).af. | | 11 |  |
| limit 7 to (english language and yr="2009 - 2018") | | 9 |  |

**IEEExplore:**

(((social robot OR social robots OR socially assistive robot OR socially assistive robots) AND mental health OR counseling OR therapy OR psychotherapy) NOT ((infant OR child OR adolescent) NOT adult))

Limit (English language and yr= “2009-2018”)
